# Right ventricular injury in patients with COVID-19-related ARDS eligible for ECMO support: a multicenter retrospective study

**DOI:** 10.1186/s13613-024-01256-8

**Published:** 2024-03-26

**Authors:** Matthieu Petit, Misylias Bouaoud, Edouard Jullien, Adrien Joseph, Bruno Evrard, Cyril Charron, Anousone Daulasim, Annick Legras, Maeva Gourraud, Marine Goudelin, Philippe Vignon, Antoine Vieillard-Baron

**Affiliations:** 1 Medical Intensive Care Unit, Ambroise Paré Hospital, APHP, 9 Avenue Charles de Gaulles, Boulogne-Billancourt, France; 2 Paris-Saclay University, UVSQ, Inserm, CESP, 94807 Villejuif, France; 3 Intensive Care Unit, University Hospital of Tours, Tours, France; 4 Medical-Surgical Intensive Care Unit and Inserm CIC 1435, Dupuytren Teaching Hospital, 87000 Limoges, France

**Keywords:** Acute respiratory distress syndrome, COVID-19, Right ventricle, Extracorporeal membrane oxygenation

## Abstract

**Background:**

Coronavirus disease 2019 (COVID-19)-related acute respiratory distress syndrome (ARDS) is associated with high mortality. Extracorporeal membrane oxygenation (ECMO) has been proposed in this setting, but optimal criteria to select target patients remain unknown. Our hypothesis is that evaluation of right ventricular (RV) function could be helpful. The aims of our study were to report the incidence and outcomes of patients eligible for ECMO according to EOLIA criteria, and to identify a subgroup of patients with RV injury, which could be a target for ECMO.

**Methods:**

Retrospective observational study involving 3 French intensive care units (ICUs) of teaching hospitals. Patients with confirmed SARS-CoV-2 infection between March 2020 and March 2021, presenting ARDS and with available echocardiography, were included. Patients were classified in three groups according to whether or not they met the EOLIA criteria and the presence of RV injury (RVI) (“EOLIA −”, “EOLIA + RVI −” and “EOLIA + RVI + ”). RVI was defined by the association of RV to left ventricular end-diastolic area ratio > 0.8 and paradoxical septal motion. Kaplan–Meier survival curves were used to analyze outcome as well as a Cox model for 90 day mortality.

**Results:**

915 patients were hospitalized for COVID-19, 418 of them with ARDS. A total of 283 patients with available echocardiography were included. Eighteen (6.3%) patients received ECMO. After exclusion of these patients, 107 (40.5%) were classified as EOLIA −, 126 (47.5%) as EOLIA + RVI −, and 32 (12%) as EOLIA + RVI + . Ninety-day mortality was 21% in the EOLIA-group, 44% in the EOLIA + RVI-group, and 66% in the EOLIA + RVI + group (*p* < 0.001). After adjustment, RVI was statistically associated with 90-day mortality (HR = 1.92 [1.10–3.37]).

**Conclusions:**

Among COVID-19-associated ARDS patients who met the EOLIA criteria, those with significant RV pressure overload had a particularly poor outcome. This subgroup may be a more specific target for ECMO. This represented 12% of our cohort compared to 60% of patients who met the EOLIA criteria only. How the identification of this high-risk subset of patients translates into patient-centered outcomes remains to be evaluated.

## Background

Severely ill coronavirus disease 2019 (COVID-19) patients often develop acute respiratory distress syndrome (COVID-19-associated ARDS) [1] and circulatory failure [2, 3]. COVID-19-associated ARDS is associated with high mortality, and extracorporeal membrane oxygenation (ECMO) has been used in this indication [4–6] with various impacts on the outcome [4, 7]. A few observational studies have reported that venovenous (VV) ECMO reduces mortality [8, 9], essentially if patients are treated in high-volume centers [9]. The French authorities recommend the use of EOLIA (ECMO to Rescue Lung Injury in Severe ARDS) [10] criteria to select patients eligible for ECMO. However, in a period of low resources due to the high number of critically ill patients, optimal selection of patients who can benefit from the technique is fundamental [11]. A large number of mortality prediction models have been developed for ECMO patients, but they are unsuitable to provide decision support as they were developed in ECMO patients only, and the decision to start ECMO had already been taken [12].

In a large retrospective international cohort of critically ill patients with COVID-19, Huang et al. reported a 19% incidence of severe right ventricular (RV) overload which was independently associated with mortality [13]. ECMO controls factors that risk worsening RV function and improves RV function when already impaired [14].

We hypothesized that adding information about respiratory and RV function to blood gases could help improve selection of patients at high risk of mortality, in accordance with our recent study performed in a large cohort of patients with moderate to severe non-COVID-19-associated ARDS [15].

In a multicenter cohort of COVID-19-associated ARDS, we report the incidence of patients potentially eligible for VV ECMO according to EOLIA criteria, as well as their outcome, and identify a specific subgroup with a remarkably high mortality, which could best benefit from VV ECMO.

## Methods

### Study design

This was a retrospective observational study involving 3 intensive care units (ICUs) of tertiary teaching hospitals in France. Participating centers all performed critical care echocardiography for RV function evaluation and restricted indications for VV ECMO according to their standards of care.

Consecutive patients with confirmed SARS-CoV-2 infection between March 2020 and March 2021 admitted to the ICU for invasive mechanical ventilation with a diagnosis of ARDS using Berlin definition [16] were screened. The period of observation was 7 days after intubation and patients with no available echocardiography during this period were excluded. The study protocol was approved by the ethics committee of the French Society of Intensive Care Medicine (ref SRLF CE 21–98).

### Patient characteristics and clinical evaluation

We calculated the Sequential Organ Failure Assessment (SOFA) score [17] at admission and the simplified acute physiology score II (SAPS II) [18]. Pre-intubation characteristics, in particular history of vaccination, body mass index, co-morbidities, interval between onset of symptoms and ICU admission and between ICU admission and intubation, were collected. We also reported the use of dexamethasone, anti-IL-6-receptor, prone position, nitric oxide inhalation, and VV ECMO, as well as clinical and respiratory characteristics (time since intubation, respiratory settings with tidal volume, respiratory rate, positive end-expiratory pressure [PEEP], plateau pressure, driving pressure, PaO_2_, PaCO_2_, FiO_2_) at the time the patient met for the first time the EOLIA criteria, or at the time of the worst PaO_2_/FiO_2_ if never met, during the first 7 days of mechanical ventilation. EOLIA criteria were defined as follows: PaO_2_/FiO_2_ of < 50 mmHg for > 3 h or PaO_2_/FiO_2_ < 80 mmHg for > 6 h or an arterial blood pH < 7.25 with a PaCO_2_ > 60 mmHg despite ventilator optimization. In-ICU, in-hospital, and 90 day mortality were recorded.

### Echocardiography

Echocardiography was performed by either a transesophageal or transthoracic approach according to the usual practice of the centers. Patients were all intubated, sedated, and adapted to the ventilator. We focused on the echocardiography performed within 24 h when patients met the EOLIA criteria, or the worst PaO_2_/FiO_2_ when EOLIA criteria were not met, during the first 7 days of invasive mechanical ventilation. Conventional parameters of RV and left ventricular (LV) function traditionally reported in ARDS patients [19, 20] were systematically obtained. Briefly, we measured LV ejection fraction as a marker of LV systolic function, and maximal velocity of mitral inflow (E and A waves) as a marker of LV diastolic function and filling pressure. RV function was evaluated by the RV/LV end-diastolic area (EDA) ratio, the fractional area change (calculated as RV EDA minus end-systole area divided by EDA), and the septal motion (whether or not paradoxical). Pulmonary acceleration time was also recorded as a marker of pulmonary hypertension, as well as the velocity–time integral (VTI) in the LV outflow track (aortic VTI).

### Statistical analysis

Baseline characteristics were reported as median [interquartile range] and *n* (%) for quantitative and qualitative variables, respectively. Quantitative variables were compared using nonparametric tests, the Mann–Whitney test, or the Kruskal–Wallis test, as appropriate. Qualitative variables were compared using Pearson’s Chi-square test or Fisher’s exact test, as appropriate.

**Fig. 1:**
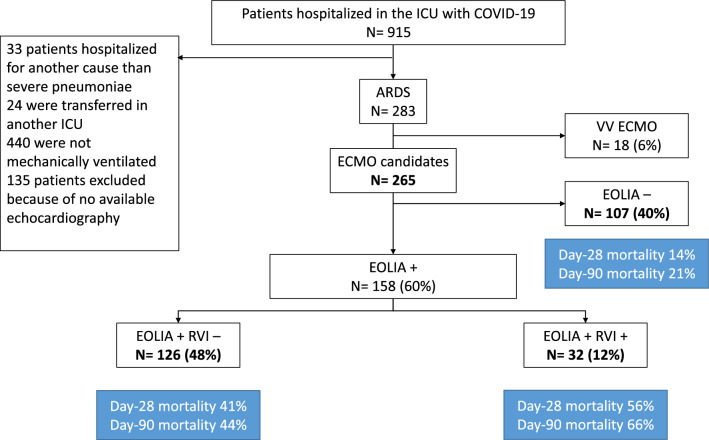
Study flowchart. ICU: Intensive Care Unit, ARDS Acute Respiratory Distress Syndrome, VV ECMO VenoVenous Extracorporeal Membrane Oxygenation, RVI Right Ventricular Injury

Missing data were reported. Determination of different subgroups was performed in patients in whom ECMO was not implemented. Accordingly, 18 patients were excluded from this analysis. Subgroups were defined according to whether or not the EOLIA criteria were met, and the presence of RV injury (RVI), leading to 3 different subgroups, “EOLIA -”, “EOLIA + RVI −” and “EOLIA + RVI + ” (Fig. 1). RVI was defined as the presence of significant RV dilatation (RVEDA/LVEDA > 0.8) and paradoxical septal motion. The cutoff for RV dilatation was chosen based on the median in the cohort. Survival analysis was compared between groups using the Kaplan–Meier method to estimate cumulative death rates, which were then compared by the log rank test. Risk factors associated with 90 day mortality in patients presenting EOLIA criteria were analyzed using a multivariable Cox model. Briefly, the variable of interest, i.e., RVI, and all variables known to have an impact on mortality in COVID-19-associated ARDS and associated with survival in univariate analysis, were included. The effects of the variables were reported in hazard ratios with 95% confidence intervals and reported on a Forest plot.

A *p* value lower than 0.05 was considered significant. All statistical analyses were performed using R Studio (*R* version 4.2.0, The R Foundation for statistical computing).

## Results

### Patients’ characteristics at admission

Between March 2020 and 2021, 915 patients were hospitalized in the participating ICUs for COVID-19, 418 of them with ARDS. A total of 283 patients with available echocardiography were enrolled in the study (Fig. 1). Their baseline characteristics on ICU admission are given in Table 1. Median age was 67 [59;72] years, SOFA score was 4 [3;5], and SAPS II 37 [31;43]. During these first three waves, only 14 patients were vaccinated against SARS-CoV-2. One hundred and eighty-three (65%) patients were treated with corticosteroids and 21 (7.4%) with anti-IL6-receptor. Median time from ICU admission to intubation was 1 [1;3] day. Most patients (80%) were prone positioned within 7 days following tracheal intubation.

**Table 1:** Baseline characteristics of patients on ICU admission Categorical variables are expressed as number (%) and continuous variables as median [interquartile ranges]

Variable	Available/ total (%)	All patients *n* = 283
Age (years)	283/283 (100)	67 [59;72]
Sex (male), *n* (%)	283/283 (100)	202 (71)
Body mass index (kg/m^2^)	283/283 (100)	29.4 [26.0;34.3]
History of cardiac disease, *n* (%)	283/283 (100)	38 (13)
History of chronic respiratory disease, *n* (%)	283/283 (100)	9 (3)
Diabetes mellitus, *n* (%)	283/283 (100)	93 (33)
Cirrhosis, *n* (%)	283/283 (100)	4 (1)
Solid organ malignancy, *n* (%)	283/283 (100)	12 (4)
Hematological malignancy, *n* (%)	283/283 (100)	14 (5)
Hypertension, *n* (%)	283/283 (100)	159 (56)
Immunosuppression, *n* (%)	283/283 (100)	43 (18)
SARS-CoV-2 vaccination, *n* (%)	191/283 (67)	14 (7)
SOFA score on ICU admission	283/283 (100)	4 [3;5]
SAPS II	283/283 (100)	37 [31;43]
Onset of symptoms to ICU admission (days)	283/283 (100)	8 [6;11]
PaO_2_/FiO_2_ on admission (mmHg)	281/283 (99)	93 [73;120]
PaCO_2_ on admission (mmHg)	281/283 (99)	34 [31;38]
Respiratory rate on admission (cycles/min)	275/283 (97)	33 [29;38]
Use of corticosteroids, *n* (%)	283/283 (100)	183 (65)
Use of anti-IL6-R, *n* (%)	283/283 (100)	21 (7)
Modality of oxygenation before invasive mechanical ventilation, *n* (%)	283/283 (100)	
*Standard oxygen therapy*		32 (11)
*High-flow oxygen therapy*		244 (86)
*Noninvasive ventilation*		7 (3)
ICU admission to intubation (days)	283/283 (100)	1 [1;3]

*SOFA: Sequential Organ Failure Assessment*

### Patients’ characteristics on meeting EOLIA criteria (or at the worst PaO_2_/FiO_2_) and outcome

Respiratory and echocardiographic parameters are listed in Table 2**.** Patients were ventilated with a median tidal volume of 6.3 [5.9;7] mL/kg of predicted body weight, with a plateau pressure of 27 [24;30] cmH_2_O, a driving pressure of 16 [13;19] cmH_2_O, and a PEEP of 10 [8;13] cmH_2_O. Most patients (76%) experienced RV dilatation and median RV/LV EDA was 0.74 [0.59;0.88]. Paradoxical septal motion was identified in 93 (39%) patients. Median ICU stay was 20 [12;36] days, and median time on invasive ventilation was 15 [9;30] days. In-ICU, in-hospital, and 90-day mortalities were 36.0%, 36.4%, and 37.5%, respectively.

**Table 2:** Clinical characteristics and echocardiographic findings at the time the patient met the EOLIA criteria or of the worst PaO_2_/FiO_2_ within the first 7 days after tracheal intubation

Variable	*n* (available/total)	All patients *n* = 283
Clinical characteristics		
Time from intubation to worst PaO_2_/FiO_2_ (days)	283/283 (100)	2 [0;4]
Vasopressor support, *n* (%)	283/283 (100)	95 (34)
Inotropic support, *n* (%)	283/283 (100)	4 (1)
PaO_2_/FiO_2_ (mmHg)	283/283 (100)	78 [56;94]
PaCO_2_ (mmHg)	283/283 (100)	52 [45;62]
pH	283/283 (100)	7.33 [7.32;7.38]
Plateau pressure (cmH_2_O)	263/283 (93)	27 [24;30]
PEEP (cmH_2_O)	283/283 (100)	10 [8;13]
Driving pressure (cmH_2_O)	263/283 (93)	16 [13;19]
Respiratory rate (cycle/min)	283/283 (100)	28 [24;32]
Tidal volume (mL/kg PBW)	283/283 (100)	6.3 [5.9;7]
Central venous pressure (mmHg)	108/283 (38)	10 [8;12]
Echocardiographic findings		
LVEF (%)	231/283 (82)	59 [52;66]
LVOT VTI (cm)	230/283 (81)	20.8 [17;24.2]
E/A ratio	214/283 (76)	0.95 [0.77;1.17]
RVEDA/LVEDA	232/283 (82)	0.74 [0.59;0.88]
Paradoxical septal motion, *n* (%)	236/283 (83)	93 (39)
RV fractional area change (%)	222/283 (78)	42 [33;48]
Pulmonary acceleration time (ms)	181/283 (64)	83 [66;100]

Categorical variables are expressed as number (%) and continuous variables as median [interquartile ranges]. PEEP Positive End-Expiratory Pressure, PBW Predicted Body Weight, LVEF Left Ventricular Ejection Fraction, LVOT VTI Left Ventricular Outflow Tract Velocity–Time Integral, RV Right Ventricular

### Characteristics of the different subgroups with their respective outcome and factors associated with 90 day mortality

Eighteen patients (6%) received VV ECMO with a worst PaO_2_/FiO_2_ and concomitant PaCO_2_ values of 59 [49;72] mmHg and 65 [54;71] mmHg, respectively. Their in-hospital mortality was 44%. After exclusion of these patients, 158/265 (60%) patients were eligible for ECMO according to EOLIA criteria within 7 days following tracheal intubation. Among them, 126 were classified in the EOLIA + RVI– subgroup and 32 in the EOLIA + RVI + subgroup (Fig. 1, Table 3). No difference in shock and in norepinephrine dose was observed between subgroups.

**Table 3:** Characteristics of the 265 patients included in the subgroup analysis according to their subgroups

Variable	EOLIA−*n* = 107	EOLIA + RVI−*n* = 126	EOLIA + RVI + *n* = 32	*p*
Baseline characteristics				
Sex (male), *n* (%)	71 (66.4)	92 (73.0)	25 (78.1)	0.340
Age (years)	69 [60, 73]	68 [60, 72]	69 [61, 74]	0.662
Body mass index (kg/m^2^)	28.00 [25.00, 33.00]	30.71 [26.52, 35.21]	31.42 [27.92, 33.41]	0.009
SOFA score at admission	4 [3, 5]	4 [3, 5]	4 [4, 5]	0.652
SAPS II	36 [29, 42]	40 [35, 47]	36 [31, 41]	0.001
Chronic respiratory failure, *n* (%)	3 (2.8)	5 (4.0)	1 (3.1)	0.884
Immunosuppression, *n* (%)	22 (20.6)	18 (14.3)	1 (3.1)	0.050
ICU admission to intubation (days)	1 [0, 3]	1 [0, 2]	2 [1, 4]	0.156
Intubation to compliance with EOLIA criteria or worst PaO_2_/FiO_2_ (days)	1 [0, 2]	2 [0,4]	2 [1, 4]	0.002
Use of prone positioning (%)	76 (71)	105 (83)	26 (81)	0.069
Respiratory parameters				
PaO_2_/FiO_2_ (mmHg)	100 [91, 115]	59 [51, 70]	59 [44, 69]	< 0.001
PaCO_2_ (mmHg)	48 [43, 55]	54 [46, 64]	60 [52, 76]	< 0.001
pH	7.36 [7.30, 7.41]	7.31 [7.25, 7.36]	7.25 [7.21, 7.33]	< 0.001
Plateau pressure (cmH_2_O)	27 [23, 30]	27 [24, 29]	28 [25, 30]	0.484
PEEP (cmH_2_O)	12 [10, 14]	10 [7, 12]	9 [7, 12]	< 0.001
Driving pressure (cmH_2_O)	14 [12, 18]	17 [15, 20]	18 [14, 22]	< 0.001
Respiratory rate (cycle/min)	30 [25, 32]	26 [22, 30]	30 [26, 34]	0.001
Echocardiographic findings				
Shock (%)^a^	29	46	11	0.471
LVEF (%)	59 [50, 65]	60 [52, 66]	62 [55, 65]	0.522
LVOT VTI (cm)	20 [16, 24]	21 [18, 24]	18 [15, 25]	0.177
RVEDA/LVEDA	0.74 [0.60, 0.85]	0.70 [0.58, 0.81]	1.00 [0.93, 1.18]	< 0.001
RV fractional area change (%)	44 [36, 51]	43 [34, 48]	36 [26, 43]	0.007
Pulmonary acceleration time (ms)	91 [72, 109]	83 [60, 100]	69 [50, 80]	< 0.001
Paradoxical septal motion, *n* (%)	40 (37.7)	21 (17.4)	32 (100.0)	< 0.001

Patients with ECMO (*n* = 18) were excluded from the analysis

Categorical variables are expressed as number (%) and continuous variables as median [interquartile range]

SOFA Sequential Organ Failure Assessment, SAPS II Simplified Acute Physiology Score, PEEP Positive End-Expiratory Pressure, LVEF Left Ventricular Ejection Fraction, LVOT VTI Left Ventricular Outflow Tract Velocity–Time Integral, RVEDA Right Ventricular End-Diastolic Area, LVEDA Left Ventricular End-Diastolic Area

^a^Shock was defined by the need of norepinephrine infusion

In the EOLIA- group, patients had lower severity according to blood gases and respiratory mechanics with a moderate RVI reflected by a median RVEDA/LVEDA of 0.74 [0.66;0.85] and paradoxical septal motion in 40% of cases. In the EOLIA + RVI + group, patients displayed the highest severity of RVI with median RVEDA/LVEDA of 1 [0.93, 1.18] and the lowest pulmonary acceleration time (69 [50, 80] ms). By definition, all had paradoxical septal motion. In contrast, in the EOLIA + RVI− group, while the patients had similar respiratory severity, RVI was less severe (RVEDA/LVEDA of 0.70 [0.58, 0.81]), median pulmonary acceleration time was 83 [60, 100] ms, and paradoxical septal motion was seen in 17% of cases).

**Fig. 2:**
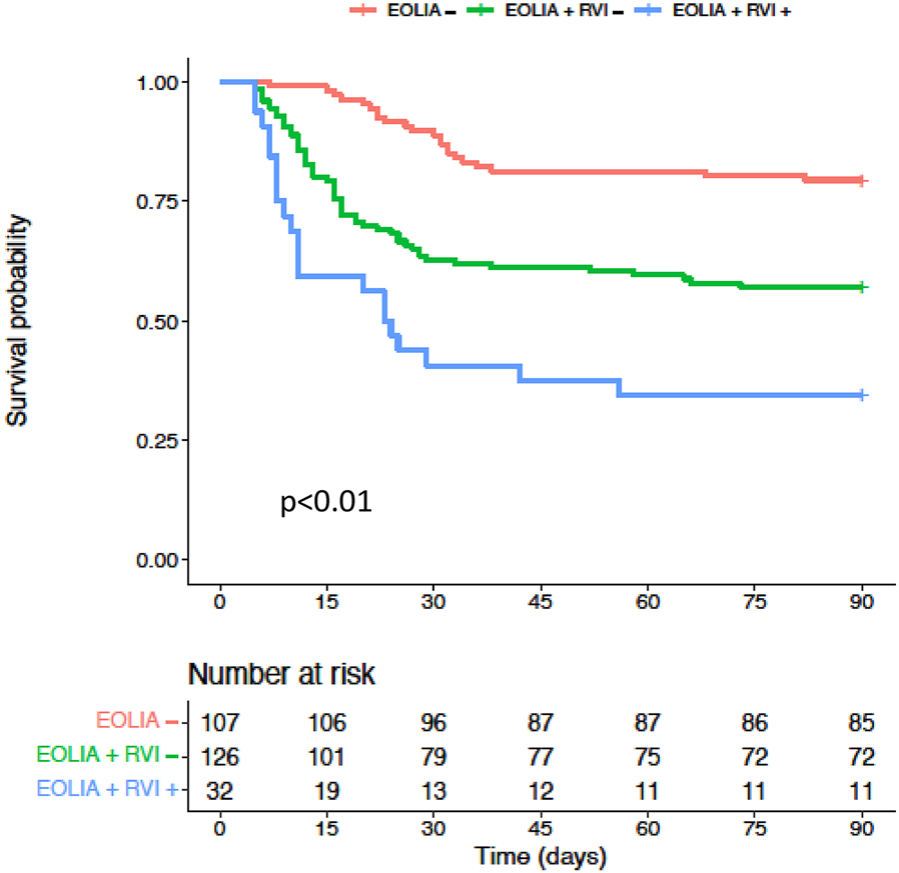
Kaplan–Meier survival estimates among the three subgroups. *p* value was obtained with a log rank test. RVI Right Ventricular Injury

**Fig. 3:**
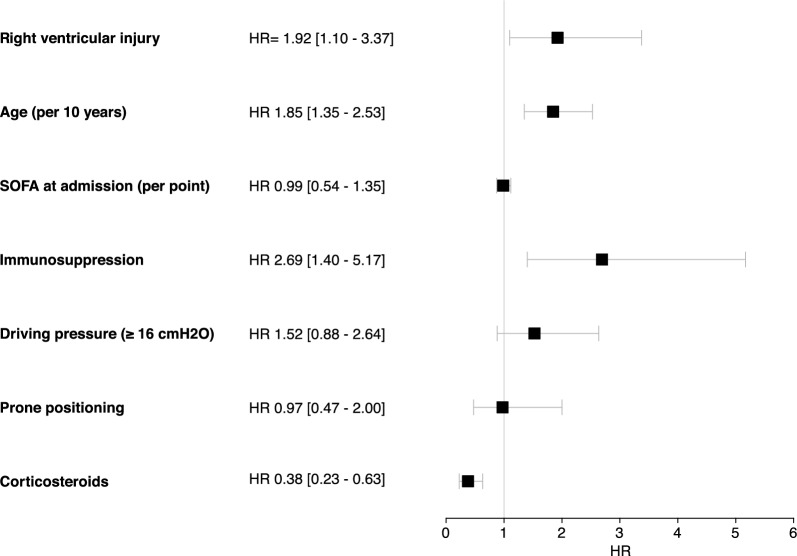
Risk factors for death at day 90 among patients reaching EOLIA criteria within 7 days after intubation. SOFA Sequential Organ Failure Assessment

Survival curves among the three groups are shown in Fig. 2. At day 90, mortality was 21% in the EOLIA− group, 44% in the EOLIA + RVI− group, and 66% in the EOLIA + RVI + group (*p* < 0.001). In a multivariable model, including age, SOFA score at admission, immunosuppression, driving pressure, prone positioning and corticosteroids, RVI was statistically associated with 90-day mortality (HR = 1.92 [1.10–3.37]) (Fig. 3).

## Discussion

In a large cohort of patients with COVID-19-associated ARDS, an approach combining clinical, respiratory, and echocardiographic parameters characterized 3 different subgroups within 7 days after tracheal intubation. A specific subgroup of patients with the most severe respiratory impairment and RV pressure overload (as shown by significant increase in RV size and paradoxical septal motion), had a significantly higher mortality compared to the other patients.

How we define the target population that could best benefit from VV ECMO is crucial when a pandemic and ICU surge lead to resource constraints. When only based on blood gases and EOLIA criteria [10], a large proportion of our patients (60%) were potentially eligible for ECMO. Conversely, it was in only 8.9% in our previous cohort of 752 patients with moderate to severe non-COVID-19-associated ARDS [15]. This well reflects the particular severity of lung injury in patients ventilated for COVID-19-related pneumonia. Moreover, in-hospital mortality of the 158 patients meeting EOLIA criteria within 7 days following tracheal intubation but managed without ECMO in the present cohort was similar to that of the 18 patients managed with ECMO (47% versus 44%, respectively). It was also similar to the in-hospital mortality rate previously reported in patients managed with ECMO in the international Extracorporeal Life Support Organization registry which ranged between 38% and 59%, according to the time of ECMO implantation (before/after May 1, 2020) and to early or late-adopting centers in a much younger population than ours [7]. In this latter series, only 60% of patients were turned prone compared to 80% in our study population.

Our results suggest that the target population could be patients presenting the EOLIA criteria and displaying RVI (12% of the population) combining very severe illness according to blood gases and respiratory mechanics with remarkable RV pressure overload. Those patients had the highest mortality (90 day mortality of 66%). Evrard et al. [21] have shown that ventilated patients with COVID-19-associated ARDS exhibited worsening of respiratory function when they developed RVI. Importantly, VV ECMO reduces RV afterload by rapidly and efficiently improving blood oxygenation and decarboxylation [14, 20], and by allowing ultra-protective ventilation with a more pronounced reduction of plateau and driving pressures [22]. A recent study in a small cohort of 15 patients with COVID-19-associated ARDS found that 47% of them exhibited severe RV pressure overload, and that RV function as well as circulatory failure rapidly improved within 24 h following ECMO [14].

In a recent systematic review and meta-analysis among patients receiving ECMO for COVID-19, driving pressure higher than 16 cmH_2_O was associated with mortality (high certainty), whereas pre-cannulation renal replacement therapy was not (low certainty) [23]. However, we did not find in our cohort an association between driving pressure and mortality.

Cain et al. found that RV dysfunction on VV ECMO was independently associated with survival and may occur or persist after VV ECMO initiation, even after controlling for all risk factors of pulmonary hypertension [24]. While there is no actual recommendation for the treatment of such a condition, prone positioning on ECMO can be tested as it can improve respiratory system compliance [25], and then hemodynamics. Inhaled nitric oxide can also be considered [26, 27]. Finally, veno-pulmonary ECMO with the ProtekDuo cannula has the interesting advantage of combining both cardiocirculatory support (for the right ventricle) and oxygenation without recirculation. A few studies have reported successful outcomes in COVID-19-associated ARDS [28]. However, the device is not available everywhere and more studies are needed to evaluate its potential benefit.

Our study suffers from limitations. First, the impact of the pandemic on the burden of healthcare workers precluded us from conducting a prospective study. Therefore, a large number of ventilated patients (*n* = 135) were excluded from the present study because echocardiography was not available, resulting in a selection bias which could alter our results. Timing of ICU admission and high strain could influence the decision to perform echocardiography. However, the way we selected patients could lead us to include the most severely ill ones, those who underwent echocardiography, thus reinforcing our results. Second, the timing of echocardiographic assessment was not standardized among centers, and timing between echocardiography and the first session of prone positioning was not collected. However, RVI was assessed within 24 h of compliance with the EOLIA criteria, during which ECMO implantation can be discussed. Third, missing data forced us to exclude some variables from the analysis because they were rarely recorded, as central venous pressure, which is of importance in evaluating RVI. Fourth, the study was performed in patients admitted to centers in which critical care echocardiography is usual practice for most physicians, and this could limit the external validity of our results. Indeed, applying our suggested approach to a target ECMO population means that intensivists are trained enough to evaluate RV pressure overload by this technique. Finally, due to the retrospective nature of the cohort, the reason for the clinical decision whether or not to initiate ECMO cannot be captured. Decision-making is complex and factors other than classical ECMO criteria can intervene. Therefore, we chose to exclude patients already treated with ECMO from the survival analysis, but it remains to be demonstrated prospectively how our approach can positively affect the therapeutic strategy and outcome of patients with ARDS.

In conclusion, we identified a subgroup of patients with significant RV pressure overload and respiratory impairment with a poor outcome, which could help physicians determine when and in whom to initiate ECMO. This represented 12% of our cohort compared to 66% of patients who met the EOLIA criteria. How the identification of this high-risk subset of patients translates into patient-centered outcomes will need to be evaluated in the future.

For further studies focusing on the use of ECMO in ARDS, evaluation of RV function before cannulation should be performed prospectively to confirm or not the impact of RVI on prognosis before designing a randomized control trial comparing a strategy of initiation of ECMO on the basis of RVI criteria to a more conventional indication.

## Data Availability

The data set used during this study is available from the corresponding author on reasonable request.

## Abbreviations

ARDS: Acute Respiratory Distress Syndrome
COVID-19: Coronavirus disease 2019
ECMO: ExtraCorporeal Membrane Oxygenation
EDA: End-Diastolic Area
ICU: Intensive Care Unit
LV: Left Ventricular
PEEP: Positive End-Expiratory Pressure
RV: Right Ventricular
RVI: Right Ventricular Injury
SOFA: Sequential Organ Failure Assessment
SAPS II: Simplified Acute Physiology Score II
VTI: Velocity–Time Integral
